# Ultrasound Guidance in Performing a Tendoscopic Surgery to Treat Posterior Tibial Tendinitis: A Useful Tool?

**DOI:** 10.1155/2016/7262413

**Published:** 2016-07-12

**Authors:** Akinobu Nishimura, Shigeto Nakazora, Aki Fukuda, Ko Kato, Akihiro Sudo

**Affiliations:** ^1^Department of Orthopaedic and Sports Medicine, Graduate School of Medicine, Mie University, 2-174 Edobashi, Tsu, Mie 514-8507, Japan; ^2^Department of Orthopaedic Surgery, Suzuka Kaisei Hospital, 112 Kou, Suzuka, Mie 513-8505, Japan; ^3^Department of Orthopaedic Surgery, Graduate School of Medicine, Mie University, 2-174 Edobashi, Tsu, Mie 514-8507, Japan

## Abstract

A 25-year-old man with a pronation-external rotation type of fracture was surgically treated using a fibular plate. Five years later, he underwent resection of bone hyperplasia because of the ankle pain and limitation of range of motion. Thereafter, the left ankle became intermittently painful, which persisted for about one year. He presented at the age of 43 with persistent ankle pain. Physical and image analysis findings indicated a diagnosis of posttraumatic posterior tibial tendinitis, which we surgically treated using tendoscopy. Endoscopic findings showed tenosynovitis and fibrillation on the tendon surface. We cleaned and removed the synovium surrounding the tendon and deepened the posterior tibial tendon groove to allow sufficient space for the posterior tibial tendon. Full weight-bearing ambulation was permitted one day after surgery and he returned to his occupation in the construction industry six weeks after surgery. The medial aspect of the ankle was free of pain and symptoms at a review two years after surgery. Although tendoscopic surgery for stage 1 posterior tibial tendon dysfunction has been reported, tendoscopic surgery to treat posttraumatic posterior tibial tendinitis has not. Our experience with this patient showed that tendoscopic surgery is useful not only for stage 1 posterior tibial dysfunction, but also for posttraumatic posterior tibial tendinitis.

## 1. Introduction

Tendoscopy of the tendons around the posterior ankle joint can be technically demanding but can offer a unique perspective of pathological processes. Some tendoscopic procedures have recently been applied to the foot and ankle [1]. An endoscopic tendon procedure was first described by Wertheimer et al. [2] in 1995. van Dijk et al. described the application of tendoscopic surgery for the posterior tibial [3] and peroneal tendons [4]. Tendoscopic synovectomy can provide good control of stage 1 posterior tibial tendon dysfunction (PTTD) [5, 6]. On the other hand, damage to the posterior tibial tendon and/or its retinacula can be associated with ankle fractures [7, 8]. Stenosing tenosynovitis may follow ankle fractures. Here, we applied tendoscopic surgery to treat posttraumatic posterior tibial tendinitis.

## 2. Case Report

A 43-year-old male construction worker presented with left medial ankle pain. He fell and twisted the left ankle at the age of 25 years. Fibular fracture was diagnosed using simple X-rays and then he was admitted to the emergency department for open reduction and internal fixation using a fibular plate at a hospital near the accident location. Five years later, left ankle pain developed and the range of ankle motion became limited, so he underwent resection of bone hyperplasia. Thereafter, the left ankle became intermittently painful, which persisted for about one year.

**Figure 1:**
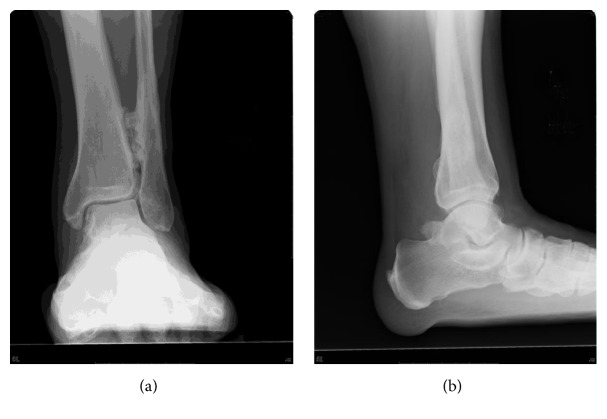
Preoperative radiographic findings. Anteroposterior (a) and lateral (b) views while standing upright.

**Figure 2:**
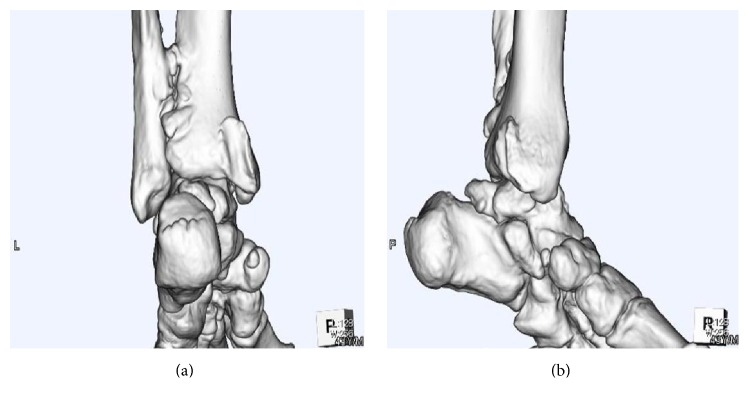
Three-dimensional computed tomography findings. Posteroanterior (a) and medial-lateral (b) views while standing upright.

**Figure 3:**
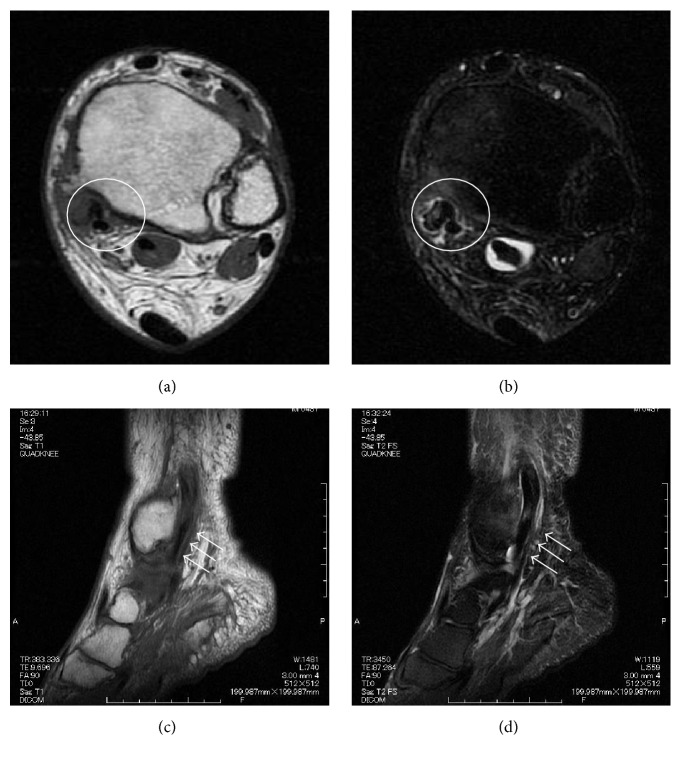
Magnetic resonance imaging (MRI). (a) T1- and (b) T2-weighted represent axial images. (c) T1- and (d) T2-weighted represent sagittal images. Circle and arrow show the posterior tibial tendon.

The initial examination at our hospital (18 years after the ankle fracture and 43 years old at this time) revealed tenderness along the posterior tibial tendon and a left ankle range of motion from 10° of dorsiflexion to 60° of plantarflexion. He was overweight at that time (his body mass index was 35.6 kg/m^2^). The left foot was relatively flat, but too much toe sign was not visible and he could perform left single-leg heel raises. X-rays revealed bone hyperplasia between the distal tibia and fibula (Figure 1). Three-dimensional computed tomography (3D-CT) revealed tibial bone hyperplasia along the posterior tibial tendon (Figure 2). The posterior tibial tendon had spotted isointensity in regions of low intensity on T1-weighted magnetic resonance (MR) images and spotted high intensity in low-intensity T2-weighted images (Figure 3). Injecting lidocaine into the posterior tibial tendon sheath relieved the pain. Thus, we diagnosed posttraumatic posterior tibial tendinitis.

**Figure 4:**
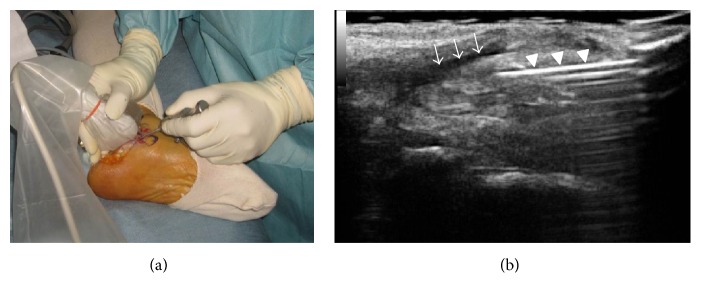
Sonographic findings. Macroscopic (a) and sonographic (b) findings. Arrows show the irrigation fluid and arrowheads show the needle.

**Figure 5:**
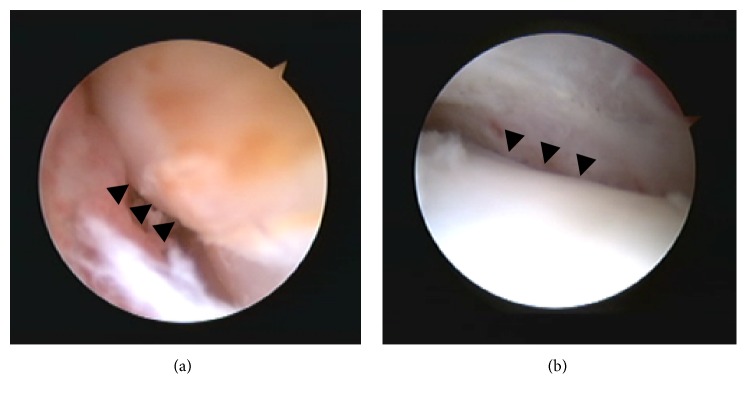
Tendoscopic findings. Before (a) and after (b) synovectomy and groove deepening. Arrowheads show the posterior tibial tendon.

We planned a tendoscopic procedure instead of an open procedure because an open procedure needs opening the tendon sheath and postoperative immobilization such as a cast or brace. The patient underwent left posterior tibial tendoscopy using a 2.5 mm 30° arthroscope under spinal anesthesia. We used the two main portals described by van Dijk in 1997 [3]. The distal portal was 2 cm below and anterior to the medial malleolus, whereas the proximal portal was 2 cm posterior and superior to the medial malleolus. An ultrasound machine (Noblus; Hitachi Aloka, Tokyo, Japan) and a high-frequency linear probe (L64 linear probe 18–5 MHz; Hitachi Aloka) are prepared with a sterile ultrasound probe cover and sterile gel. We used an ultrasonography in order to introduce a scope easily, reliably, and safely. Irrigation solution was injected into the posterior tendon sheath under long-axis view along the posterior tibial tendon of sonography just before incising the skin (Figure 4). We opened the tendon sheath via the skin incision by blunt dissection using a hemostat under sonography. Tendoscopic findings revealed tenosynovitis and fibrillation on the surface of the tendon. We cleaned and removed the synovium surrounding the tendon using an arthroscopic shaver system and a radiofrequency wand for small joints (Figure 5). Moreover, we deepened the groove for the posterior tibial tendon using a bone cutter/shaver so as the posterior tibial tendon can move smoothly. After groove deepening, there was one- or two-millimeter space around the tendon. Weight-bearing ambulation was permitted one day after surgery and the patient returned to full-time work in the construction industry six weeks after surgery. At the most recent review two years after surgery, he had no medial symptoms and he was capable of all types of heavy work despite occasional slight pain related to sinus tarsi syndrome. His preoperative and postoperative scores on the American Orthopaedic Foot and Ankle Society (AOFAS) ankle-hindfoot scale were 71 and 90 points, respectively.

Each author certifies that his or her institution approved the human protocol for this investigation and that all investigations were conducted in conformity with ethical principles of research. The patient and his family were informed that data from the case would be submitted for publication and gave their consent.

## 3. Discussion

Advances in small joint arthroscopy have led to the development of many effective arthroscopic and endoscopic procedures for foot and ankle conditions [1]. van Dijk et al. [3] first described tendoscopy of the posterior tendon to manage tendon pathology. Chow et al. [5] reported that the results of a case series of tendoscopic synovectomy for six patients with stage 1 PTTD were as good as those reported for open synovectomy [9, 10]. Lui [11] described tendoscopy-assisted posterior tibial tendon reconstruction for stage 2 PTTD, and G. Khazen and C. Khazen [6] reported a case series in which 8 (89%) of 9 patients with stage 1 PTTD reported absent or minor pain after undergoing tendoscopic synovectomy. Bulstra et al. [12] performed 16 procedures in 11 patients that included chronic synovitis (*n* = 2), screw removal from the medial malleolus (*n* = 1), and posterior ankle anatomy (*n* = 2). An irregular sliding channel was smoothed out in two patients and two asymptomatic patients underwent tenosynovectomy and tendon release. The outcome of posterior tibial tendoscopic synovectomy to treat posttraumatic tenosynovitis in our patient was successful.

The posterior tibial tendon that plays an important role in normal hindfoot function lies close to the medial malleolus and beneath the flexor retinaculum, which binds the tendon to the bone. Thus, this tendon can become injured when the ankle is fractured. Crim et al. [13] described a partly torn posterior tibial tendon in 4.2% of hindfoot fractures and that the tendon had become trapped between fracture fragments in 16.1% of them. The initial X-ray image of the ankle of our patient was unavailable, but the later X-ray suggested a relatively proximal fibula fracture and hyperossification between the distal tibia and fibula. These findings indicated a pronation-external rotation (PER) injury, which might have led to a deltoid ligament tear or medial malleolus fracture that may have bled into the posterior tendon sheath, ultimately leading to ossification of the posterior tendon sheath.

Posttraumatic and postsurgical complaints of pain at the posterior margin of the medial malleolus often pose a diagnostic and therapeutic challenge. In the absence of intra-articular pathology, adhesions and irregularities in the tendon sliding channel can be responsible for symptoms in this region. Open tendon release requires postoperative plaster immobilization with the subsequent potential for new adhesion formation [14]. Tendoscopic surgery offers the advantages of decreased morbidity, early range of motion, reduced postoperative pain, and rapid recovery [15].

Our patient had a tendoscopic surgery and it did not require opening the tendon sheath, so postoperative immobilization such as a cast or brace was unnecessary. However, because osteophytes could not be resected along with the posterior tibial tendon by tendoscopic surgery, we enlarged the tendon cavity by deepening the posterior tibial tendon groove. Since postoperative immobilization was not needed, tendon adhesion was not a concern. Tendoscopic surgery enabled range-of-motion exercises to be started immediately, which prevented scar tissue formation and adhesions. Therefore, tendoscopic surgery for this patient was effective and practical.

Tendoscopy is technically demanding. To introduce a scope into the posterior tendon sheath requires experience and finesse, and it is even more difficult in the presence of a subcutaneous irrigation fluid leak. Therefore, we used ultrasonographic guidance. We recommend using ultrasound guidance for especially unexperienced surgeons because it is an easy tool which enable a reliable insertion of an endoscope into the tendon sheath. Yamamoto et al. [16] reported using ultrasonographic assistance to approach a wrist ganglion. Although ultrasonographic resolution is limited, recent improvements in both hardware and software have made it an excellent, noninvasive, and dynamic technique for assessing the musculoskeletal system [17, 18] as it can visualize not only the posterior tibial tendon, but also vessels, nerves, and other tissues such as the long flexor muscles of the toes. These features considerably reduce the risk of serious postprocedural complications. We consider that sonography combined with endo/arthroscopy represents a useful and practical approach to soft tissue surgery.

In conclusion, ultrasonography-assisted tendoscopic synovectomy is useful for posterior tendon synovitis. Tendoscopic synovectomy requires specific skills, but it is more favorable than open synovectomy in terms of postoperative rehabilitation and cosmetic aspects. Ultrasonographic assistance also facilitates the introduction of a scope into the tendon sheath.
